# Microglia Actively Regulate the Number of Functional Synapses

**DOI:** 10.1371/journal.pone.0056293

**Published:** 2013-02-05

**Authors:** Kyungmin Ji, Gulcan Akgul, Lonnie P. Wollmuth, Stella E. Tsirka

**Affiliations:** 1 Department of Pharmacological Sciences, Stony Brook University, Stony Brook, New York, United States of America; 2 Graduate Program in Molecular and Cellular Biology, Stony Brook University, Stony Brook, New York, United States of America; 3 Department of Neurobiology and Behavior, Stony Brook University, Stony Brook, New York, United States of America; University of Nebraska Medical Center, United States of America

## Abstract

Microglia are the immunocompetent cells of the central nervous system. In the physiological setting, their highly motile processes continually survey the local brain parenchyma and transiently contact synaptic elements. Although recent work has shown that the interaction of microglia with synapses contributes to synaptic remodeling during development, the role of microglia in synaptic physiology is just starting to get explored. To assess this question, we employed an electrophysiological approach using two methods to manipulate microglia in culture: organotypic hippocampal brain slices in which microglia were depleted using clodronate liposomes, and cultured hippocampal neurons to which microglia were added. We show here that the frequency of excitatory postsynaptic current increases in microglia-depleted brain slices, consistent with a higher synaptic density, and that this enhancement ensures from the loss of microglia since it is reversed when the microglia are replenished. Conversely, the addition of microglia to neuronal cultures decreases synaptic activity and reduces the density of synapses, spine numbers, surface expression of AMPA receptor (GluA1), and levels of synaptic adhesion molecules. Taken together, our findings demonstrate that non-activated microglia acutely modulate synaptic activity by regulating the number of functional synapses in the central nervous system.

## Introduction

Microglia are the intrinsic immune cells of the central nervous system (CNS) and have long been recognized to sense CNS insults [1]. Upon activation, an event that follows CNS insults, they undergo morphological changes and release pro-inflammatory mediators such as tumor necrosis factor (TNF)-α and anti-inflammatory mediators such as interleukin-10 (IL-10) [2] while assisting in the clearance of cellular debris. It has now become appreciated, however, that microglia also survey the local microenvironment [3], [4], making direct contact with synaptic spines under non-pathological conditions (Tremblay et al., 2010). These synaptic interactions are influenced by neuronal feedback from sensory inputs or neuronal injury [5], [6]. Moreover, microglia may participate in synaptic remodeling during development [7]. In a recent report it was shown that microglia prune and define postnatal neural circuits by engulfing and removing presynaptic retinal ganglion terminals using C3- and CR3 mechanisms [8], [9].

Based on these reports it has become evident that microglia are involved in the structural plasticity of the CNS during postnatal development, but the functional significance of these events has not been addressed. Using loss- and gain-of-function approaches, we demonstrate here that sculpting and pruning, which decrease the number of synapses, restrict the frequency of synaptic currents. Moreover, we show that the microglia target glutamatergic synapses, and specifically AMPA receptors and synaptic adhesion molecules. Our results suggest that modifying or suppressing microglial function affects neuronal function and communication in the CNS.

## Materials and Methods

### Animals

C57BL/6 mice (wild type, WT), MacGreen mice, which express enhanced green fluorescent protein (EGFP) under the control of the macrophage-specific Csf1R promoter in the C57BL/6 background [10], or β-actin EGFP (C57BL/6-Tg[ACTβ-EGFP]1Osb/J; Jackson Laboratories) mice were used for these studies. Animal procedures were approved by the Stony Brook University Institutional Animal Care and Use Committee (IACUC).

### Organotypic hippocampal brain slice cultures

Organotypic hippocampal slices were prepared as described in Stoppini et al. [11]. In brief, under sterile conditions in a flow cabinet, 350 µm-thick slices were prepared from 3–4 day-old mouse pups. Each slice was immediately placed on a porous insert membrane (Millipore) at the interface of a serum culture medium: 50% minimal essential medium, 25% heat-inactivated horse serum, and 25% Hank's balanced salt solution with 25 mM glucose. Clodronate (5 µg/ml) or macrophage inhibitory factor (MIF, 100 µg/ml) were added to the slices in culture medium and were present for the entire incubation period unless otherwise indicated. Microglial morphology was evaluated by measuring cell body diameter as well as the number of primary processes emanating from the cell. The slices were kept at 34°C for 6 days in vitro (designated DIV6) or 14 days in vitro (DIV14). The media were changed twice weekly.

### Hippocampal neuronal and cortical microglial co-cultures

Hippocampal neurons were isolated from P0 C57BL/6 WT or β-actin EGFP mice as previously described [12]. In brief, the hippocampi from P0 mice were dissected and trypsinized (0.25% trypsin in HBSS) at 37°C for 15 min and then triturated with a fire-polished pasteur pipette. The cells were plated at a density of 2.0×10^4^ cells on coverslips precoated with poly-d-lysine (10 µg/ml) in 24-well plates. The primary cultures were grown in serum-free neurobasal media (Invitrogen, cat# 21103-049) with glutamine (Sigma) and B-27 supplement (Invitrogen, cat# 17504-044). The media were changed twice weekly. Immunostaining of the primary cultures for the neuron-specific nuclear protein (NeuN; Chemicon) showed that the cultures consisted of >99% neurons. Neurons were used in experiments after 19–20 DIV unless otherwise indicated.

Microglial cultures were prepared from the mixed cortical cultures of P0 MacGreen or WT mice as previously described [12]. Briefly, cortices were incubated in 0.25% trypsin-EDTA in HBSS at 37°C for 15 min and then washed with medium containing 10% FBS. To obtain a single-cell suspension, the tissue was triturated gently by pipetting through a 1 ml tip and then filtering through 40 µm nylon mesh, following which the cells were placed on poly-d-lysine-coated 10-cm culture plates at a density of 4 brains per plate. Two days before co-culturing with neurons, the microglia were recovered by shaking them off of the mixed cortical culture plates. After low-speed centrifugation, the pelleted microglia were resuspended in neurobasal media and plated onto the neurons at a 1∶5 microglia∶neuron ratio. Homogeneity of the microglia was determined by immunostaining for the microglial-specific marker, Iba-1; cell nuclei were counter-stained with 4′,6-diamidino-2-phenylindole (DAPI). To visualize exogenously added microglia, cultured microglia were labeled by the addition of 5 µg/ml tetramethylrhodamine and biotin (mini-ruby; Invitrogen) 12 h prior to placing on organotypic slices.

### Collection of media from brain slices and neuronal cultures and preparation of cell debris

Media from organotypic slices grown as described above in the absence or presence of clodronate or MIF for 6 or 14 days were collected, centrifuged for 5 min at 3000× g, and stored at −80°C. Media were also collected from primary neurons incubated for 2 days in the presence or absence of microglia. Medium from microglial cultures activated with Lipopolysaccharide (LPS) was used as the positive control for measurement of secreted TNF-α in experimental settings.

To prepare “cell debris”, primary astrocytes obtained from mixed cortical cultures were collected by trypsinization, centrifuged for 5 min at 3000× g, resuspended in HBSS buffer and sonicated. 3 µl of sonicated cell debris was plated on top of WT organotypic brain slices at DIV1.

### Replenishment of microglia to organotypic brain slices

Microglia harvested from mixed cortical cultures as above were labeled with miniruby (5 µg/ml) [13]. 800 labeled microglia in 2 µl were carefully laid on top of hippocampal slices that had been depleted of endogenous microglia by treatment with clodronate for 6 days. Clodronate was washed away prior to the addition of the labeled microglia. Control slices or slices with added microglia were maintained for an additional 8 days prior to whole-cell patch-clamping. The state of microglial activation was evaluated by measuring cell body diameter as well as the number of primary processes emanating from each cell, and by assessing the expression of inducible nitric oxide synthase (iNOS, not shown) and the levels of secreted TNF-α.

### TNF-α assay

Media from neurons cultured with or without microglia, from purified microglia, and from organotypic brain slices treated with clodronate or MIF were collected as described and assayed using a TNF-α enzyme-linked immunosorbent assay (ELISA) kit with sensitivity >1.07 pg/ml as per manufacturer's protocol (BD biosciences). ODs were determined at 450 nm. All assays were performed in triplicate.

### Immunohistochemistry

Hippocampal slices or cultured neurons were fixed with 4% PFA in PBS for 30 min. After washing in PBS, the samples were blocked with 0.2% Triton X-100 and 1% BSA in PBS and incubated with primary antibodies (listed in Table 1: anti-NeuN, anti-Iba-1, anti-CD11b, anti-GFAP, anti-synapsin I, anti-PSD95, anti-GluA1, anti-Mac-2, and LAMP-1) overnight at 4°C. To stain cell-surface GluA1, neurons were fixed under non-permeabilizing conditions by incubation in 4% paraformaldehyde/4% sucrose in PBS for 5 min at room temperature and incubated in 1% normal goat serum in PBS containing anti-GluA1 for 1 hr at RT. The slices and the cultures were incubated with Alexa Fluor488-, Alexa Fluor555- (Invitrogen), or Dylight 649- (Jackson Immunoresearch) conjugated secondary antibodies for 2 h at room temperature to enable fluorescent detection. Texas-red phalloidin (Invitrogen) was used to label F-actin according to the manufacturer's protocol. The slices and the cultures were mounted in Vectashield mounting media with DAPI. Images were visualized by confocal and 2-photon microscopy with Z-stack and 3D-reconstruction analysis. To quantify the number of synapses, synapsin I^+^ puncta and/or PSD95^+^ puncta per 10 µm were counted (n = 27–39).

**Table 1:** Summary of antibodies used for immunostaining or immunoblotting.

Antigen	Antibody	Dilution	Source	Catalog number	Method
NeuN	Mouse monoclonal	1∶1000	Chemicon	MAB377	IHC
		1∶5000			WB
Iba-1	Rabbit polyclonal	1∶1000	Wako	019-19741	IHC
		1∶5000			WB
CD11b	Rat monoclonal	1∶200	Chemicon	MAB1387Z	IHC
GFAP	Rabbit polyclonal	1∶1000	DAKO	Z0334	IHC
		1∶5000			WB
Alpha-tubulin	Mouse monoclonal	1∶7000	Upstate biotechnology	05-829	WB
Synapsin I	Rabbit polyclonal	1∶1000	Chemicon	AB1543P	IHC
PSD95	Mouse monoclonal	1∶200	NeuroMabs	75-028	IHC
GluA1	Rabbit polyclonal	1∶20	Chemicon	PC246	IHC
	Rabbit monoclonal	1∶2000	Millipore	05-855R	WB
Mac-2	Rat polyclonal	1∶500	cedarlane	CL8942AP	IHC
LAMP-1	Mouse monoclonal	1∶1000	Transduction laboratories	L72206	IHC
SynCAM-1	Mouse monoclonal	1∶500	NeuroMab	75-120	WB
N-cadherin	Mouse monoclonal	1∶10000	Transduction laboratories	610920	WB
Pan- γ-protocadherin	Mouse monoclonal	1∶500	NeuroMab	75-185	WB

Iba-1, ionized calcium binding adaptor molecule-1; GFAP, Glial fibrillary acidic protein, PSD95, postsynaptic density protein 95; IHC, immunohistochemistry; WB, Western blot.

### Western blot analysis

Tissues were lysed in ice-cold 50 mM Tris-HCl (pH 7.4) containing 1% Nonidet P-40, 0.25% Na-deoxycholate, 150 mM NaCl, and protease inhibitors cocktail (Sigma-Aldrich) using a tissue homogenizer. After incubation on ice for 30 min, the tissue lysates were centrifuged at 13000 rpm for 20 min at 4°C. The extracts were separated on a reducing 10% SDS-PAGE, blotted to PVDF membrane (Immobilon-P; Millipore), and incubated using primary antibodies (Table **1**: anti-NeuN, anti-Iba-1, anti-GFAP, and anti-GluA1, anti-synCAM1, anti-N-cadherin, and anti-pan-γ-protocadherin) overnight at 4°C. HRP-labeled mouse or rabbit secondary antibodies (Invitrogen) were applied for 1 h at RT. Protein bands were detected with ECL (Pierce Chemical Co.). After stripping, the membranes were reprobed with mouse anti-α tubulin antibody (Upstate Biotechnology).

### Whole-cell recording and data analysis

Membrane potentials or currents were recorded at room temperature (22–25°C) using an EPC 10 USB amplifier with PatchMaster software (HEKA Elektronik, Lambrecht, Germany). Recordings were low-pass filtered at 10 kHz (−3 dB) using an 8 pole low-pass Bessel filter and digitized at 20 kHz. For analysis, they were refiltered at 2 kHz using a digital Gaussian filter.

The recording pipettes (3–5 MΩ) were filled with an internal solution containing (in mM): 105 K-gluconate, 30 KCl, 3 MgCl_2_, 10 HEPES, 10 phosphocreatine, 4 Mg-ATP and 0.3 GTP, pH 7.3 (KOH), and adjusted to 305 mOsm with sucrose. Biocytin (0.2%) was included in the internal solution for post-fixation neuronal labeling. The artificial cerebral spinal fluid (ACSF) bath solution used for organotypic slices consisted of the following (in mM): 125 NaCl, 2.5 KCl, 25 glucose, 25 NaHCO_3_, 1.25 NaH_2_PO_4_, 2 CaCl_2_ and 1 MgCl_2_, and was saturated with 95% O_2_/5% CO_2_ under all conditions. The Ringer's solution for primary cultures composed of the following (in mM): 145 NaCl, 3 KCl, 10 Hepes, 2 CaCl_2_, 2 MgCl_2_, and 10 glucose (pH 7.4). All pharmacological agents were added to the external solution without substitution.

#### Membrane properties

Upon achieving whole cell, the resting membrane potential (RMP) was determined in current clamp. The amplifier mode was switched to voltage clamp with a baseline holding potential (Vh) of −70 mV. Pipette series resistance (Rs) and capacitance (Cm) were tested using the LOCKIN protocol from HEKA. The amplifier was then switched to current clamp mode, the membrane potential adjusted to approximately −70 mV, and a series of depolarizing current pulses of 1 s duration were injected with 200 ms between each stimulus. Action potentials and firing pattern of the neurons were monitored in response to depolarizing currents.

#### sEPSC/mEPSC recording

Neurons were voltage-clamped at −70 mV and membrane currents recorded in 3-minute blocks. Pipette series resistance, neuronal membrane resistance and capacitance were monitored using the HEKA LOCKIN protocol. Action potentials and GABA_A_-mediated currents were blocked using a cocktail of 1 µM TTX and 50 µM bicuculine or picrotoxin, respectively. At the end of some experiments, 10 µM CNQX was washed in to confirm that the observed synaptic events were AMPAR-mediated.

All reagents unless otherwise noted were obtained from either Sigma Aldrich Inc. (St. Louis, MO) or Tocris Cookson Inc. (Ellisville, MO).

Data analysis was performed using MiniAnalysis (Synaptosoft) and Igor Pro (WaveMetrics, Inc., Lake Oswego, OR). Results are presented as mean ± SEM (n≥5).

### Statistics

ANOVA or Student's t test was used to define statistical differences. The Tukey or Dunnett's test was used for multiple comparisons. Significance was assumed at p<0.05. All results are represented as average with error bars indicating the standard error of the mean.

## Results

**Figure 1:**
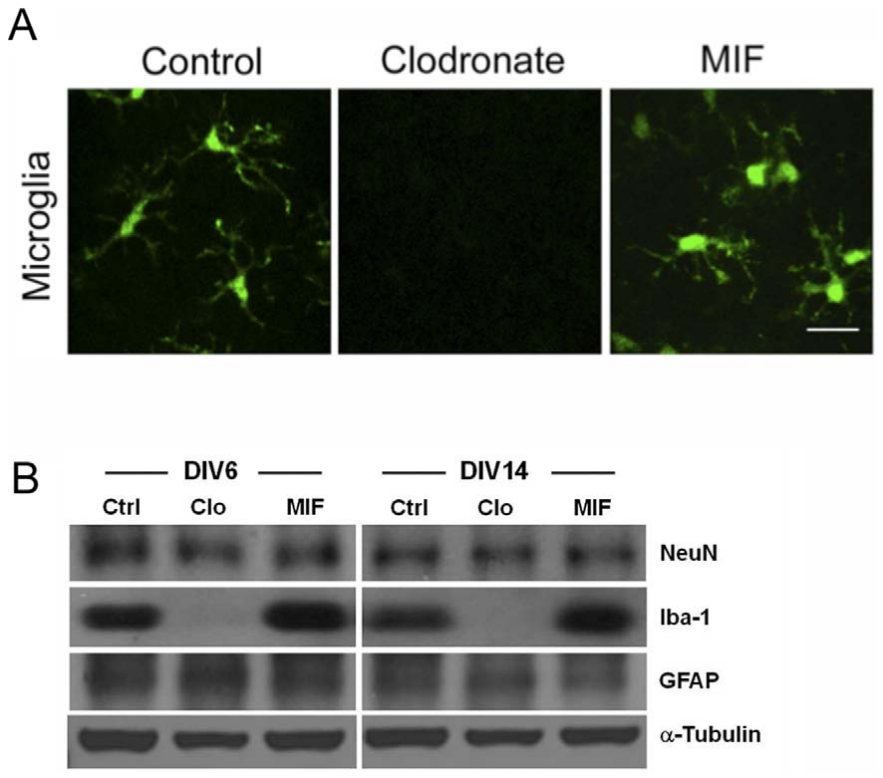
Clondronate ablates microglia in organotypic hippocampal brain slices. Hippocampal slices were obtained from MacGreen mice and were treated with clodronate (5 µg/ml) or MIF (100 µg/ml) for 6 or 14 days and examined using confocal microscopy. **A.** Microglia could not be detected in clodronate-treated slices at DIV14, whereas microglia in MIF-treated slices appear similar in morphology to microglia in control slices. Scale bar, 20 µm. **B.** Western blot analysis of NeuN, Iba-1, and GFAP. Alpha-tubulin was used as a loading control.

To examine the relationship of microglia to synaptic activity, we began by depleting microglia in organotypic hippocampal brain slices from Csf1R-EGFP (MacGreen) mice (Fig. **1A**) using clodronate encapsulated in liposomes (5 µg/ml) to elicit selective apoptosis of macrophages/microglia [14]–[16]. We also used the tripeptide macrophage/microglial inhibitory factor (MIF), Thr-Lys-Pro (100 µg/ml), to inhibit microglial activation, thus keeping them in a resting state and preventing production of TNF-α and ROS [12], [17]. Western blot analysis confirmed the presence of similar levels of NeuN (a neuronal marker) and GFAP (an astrocytic marker) in control and clodronate- and MIF-treated slices treated for 6 or 14 days in vitro (DIV), whereas Iba-1, a marker of microglia, was below the limit of detection in the clodronate-treated slices (Fig. **1B**). Immunohistochemistry using anti-NeuN and –GFAP revealed no apparent differences in the structure and density of neurons and astrocytes in the control and experimental slices, whereas the microglia were ablated in the clodronate-treated slices (Fig. **S1**). Finally, propidium iodide (PI) staining revealed increased cell death only in the clodronate-treated cultures (Fig. **S2**).

### Microglial depletion increases synaptic activity

**Figure 2:**
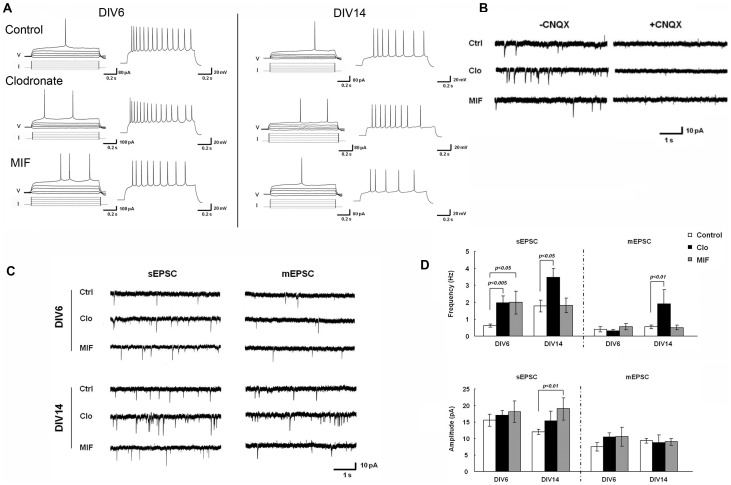
Increased mEPSC frequency in microglia-depleted organotypic hippocampal brain slices. Hippocampal organotypic brain slices obtained from MacGreen mice were treated with clodronate (5 µg/ml) or MIF (100 µg/ml) for 6 or 14 DIV. **A.** Firing patterns of CA1 pyramidal neurons in response to current injections for control and clodronate- or MIF-treated slices. **B.** CNQX, an AMPA receptor antagonist, blocks mEPSCs. Representative traces of mEPSC from control or clodronate- or MIF-treated OHBS at DIV14 in the absence or presence of 10 µM CNQX. **C.** Representative recording traces of mEPSCs from CA1 neurons in control, clodronate- or MIF-treated brain slices. **D.** Summary of mean mEPSC frequency and amplitude from control, clodronate-, or MIF-treated brain slices. Error bars represent mean ± SEM.

CA1 pyramidal neurons from organotypic slices were recorded using patch-clamp to measure synaptic properties as well as intrinsic membrane properties such as action potential shape. At the end of the recording, biocytin-labeling confirmed that the recorded cells were CA1 pyramidal neurons (data not shown). Membrane properties showed no notable difference among groups although there were some variations (Fig. **2A**, **Table 2**). Hence, as assayed by membrane properties, depletion or inactivation of microglia does not alter the general ‘health’ of CA1 pyramidal neurons.

**Table 2:** Membrane properties of CA1 pyramidal neurons in control or clodronate- or MIF-treated organotypic slices at DIV6 and 14.

	DIV6			DIV14		
Parameter	Control	Clodronate	MIF	Control	Clodronate	MIF
RMP (mV)	−67.7±1.2	−71.0±0.8*	−67.00±1.7	−63.7±0.7	−64.9±1.1	−63.7±1.6
Cm (pF)	15.3±0.7	17.4±1.4	18.0±1.8	16.4±1.3	15.6±1.5	16.1±2.8
Rm (mΩ)	243±33	212±36	186±20	188±7	169±14	171±13
Tau (ms)	3.5±0.4	3.4±0.5	3.1±0.2	3.0±0.2	2.6±0.2	2.6±0.3
Rheobase (pA)	100±5	112±8	132±8*	133±7	139±19	111±8*
Time to the first AP (ms)	438±48	531±49	525±73	561±41	312±49*	424±97
AP peak (mV)	35.6±2.0	33.0±1.8	29.0±3.6	38.8±1.7	22.0±4.0	37.1±2.7
Threshold (mV)	−44.6±0.8	−44.2±1.0	−40.3±2.2	−34.0±1.5	−38.2±3.0	−36.7±1.5
AP half-width (ms)	1.8±0.1	1.5±0.1	1.8±0.2	2.4±0.1	1.9±0.1	1.9±0.1
AHP peak (mV)	−9.2±0.5	−8.2±0.9	−10.3±1.9	−11.2±1.0	−13.1±1.9	−10.1±1.0
n	28	24	10	23	9	5

Values shown are mean ± SEM.

* , Statistically significant differences at p<0.05 when compared with the respective control at DIV6 or 14.

RMP, resting membrane potential; Cm, membrane capacitance; Rm, membrane resistance; AP, action potential; AHP, afterhyperpolarization.

To assess synaptic activity, we measured spontaneous and miniature excitatory postsynaptic currents (s- and m-EPSCs) mediated by AMPA receptors (AMPAR) and found a striking increase in the frequency of AMPAR-mediated (Fig. **2B**) mEPSCs in slices treated with clodronate. The increase was significant at DIV14 (2.2±0.9 Hz, n = 5) (p<0.01, one way ANOVA), in comparison to control slices (control DIV14, 0.6±0.1 Hz, n = 11) (Fig. **2C, 2D**), whereas mEPSC amplitude was unaffected (control: 9.4±0.8 pA; Clo: 10.0±2.3 pA; MIF: 10.2±1.0 pA) (Fig. **2C, 2D**). No statistically significant changes at DIV6 in mEPSC frequency (control DIV6: 0.4±0.2 Hz, n = 14; Clo DIV6: 0.3±0.1 Hz; MIF DIV6: 0.6±0.2 Hz, n = 9; Fig. **2D**) and amplitude (control DIV6: 7.5±1.2 pA; Clo DIV6: 10.4±1.3 pA; MIF DIV6: 10.6±2.9 pA) (p>0.05 one way ANOVA; Fig. **2D**) were detected.

**Figure 3:**
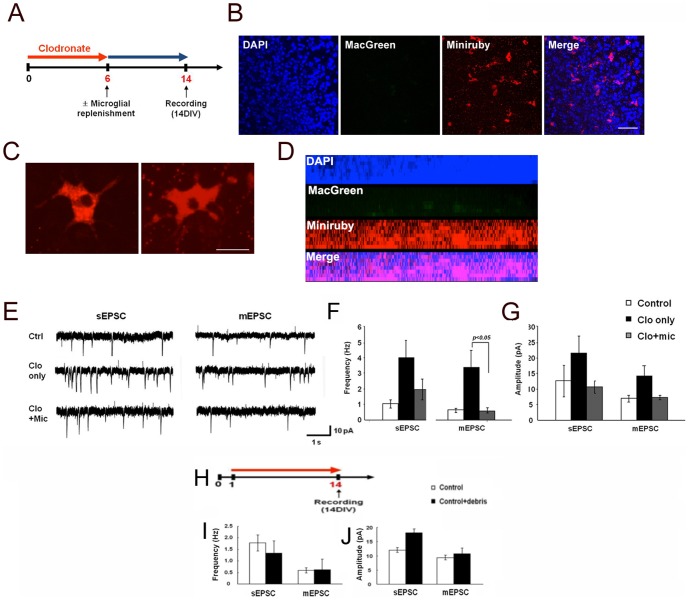
Replenishment of microglia reverses the effect of microglial depletion on organotypic brain slices. **A.** Organotypic slices obtained from MacGreen mice were treated with clodronate for 6 DIV. Clodronate was washed away and then the slices were cultured for 8 DIV in the absence of clodronate with or without primary microglia labeled with miniruby (5 µg/ml, 8×10^2^ cells in 3 µl). **B–D.** Images showing exogenously added microglia (Miniruby, red) to CA1 hippocampal slices treated with clodronate. MacGreen indicates endogenous microglia (green). Higher magnification of miniruby^+^ microglia in **C.** Side view of the z-stacking images for B. DAPI staining was used to visualize nuclei. Scale bars, 50 µm in B; 20 µm in C. **E–G.** Recording traces (**E**) and summaries of frequency (**F**) and amplitude (**G**) of sEPSCs and mEPSCs from CA1 neurons in control, clodronate-treated, and clodronate-treated with replenished microglia organotypic slices. Error bars represent mean ± SEM. **H–J.** Cellular debris does not affect sEPSC and mEPSC frequency or amplitude. **H.** Organotypic slices were incubated either without (control) or with (red line) added cellular debris (control+debris). **I, J.** Summary of sEPSC and mEPSC frequency (**I**) and amplitude (**J**) from control or cultures with added cellular debris at DIV14. Data are expressed as means ± SEM (n = 2 for each).

To test whether the increased mEPSC frequency in the microglia-depleted organotypic slices was caused by the absence of microglia, we added microglia back to the slices and examined the impact on synaptic activity (Fig. **3A**). We exposed slices to clodronate for 6 days and then either made no additional treatment (clo only) or replenished the microglia (clo+mic) using mini-ruby-stained microglia [18] and assayed the slices after 7–14 additional days of culture. After 6 days in culture, the exogenously-supplied microglia had migrated into the slice (Fig. **3B, D**) and displayed the ramified morphology characteristic of resting cells based on cell body size and the number of primary branches (Figs. **3C** and Fig. **S3**). The addition of microglia to microglial-depleted slices reverted the mEPSC frequency (clo+mic; 0.6±0.2 Hz, n = 4) back to that of the control (0.6±0.1 Hz, n = 4); both of these frequencies were significantly lower than those found in microglial-depleted slices (clo only: 3.4±1.1 Hz) (Fig. **3E, F**). Changes in mEPSP amplitude were again not statistically significant (control: 7.0±1.1 pA; clo only: 14.3±3.8 pA; clo+mic: 7.4±0.7 pA; Fig.**3G**). Hence, microglia replenishment reverses the effect of microglial depletion.

To explore whether the changes in synaptic strength might result from the presence of apoptotic debris, control brain slices were incubated with sonicated astrocytic cell debris (see Materials and Methods) for 13 days, and mEPSC recorded. mEPSC frequency and amplitude were similar to the control brain slices (Fig. **3H–J**), supporting the hypothesis that the changes in synaptic activity observed after clondronate treatment are directly due to the absence of the microglia.

### Microglia restrain synaptic frequency in primary hippocampal cultures

**Figure 4:**
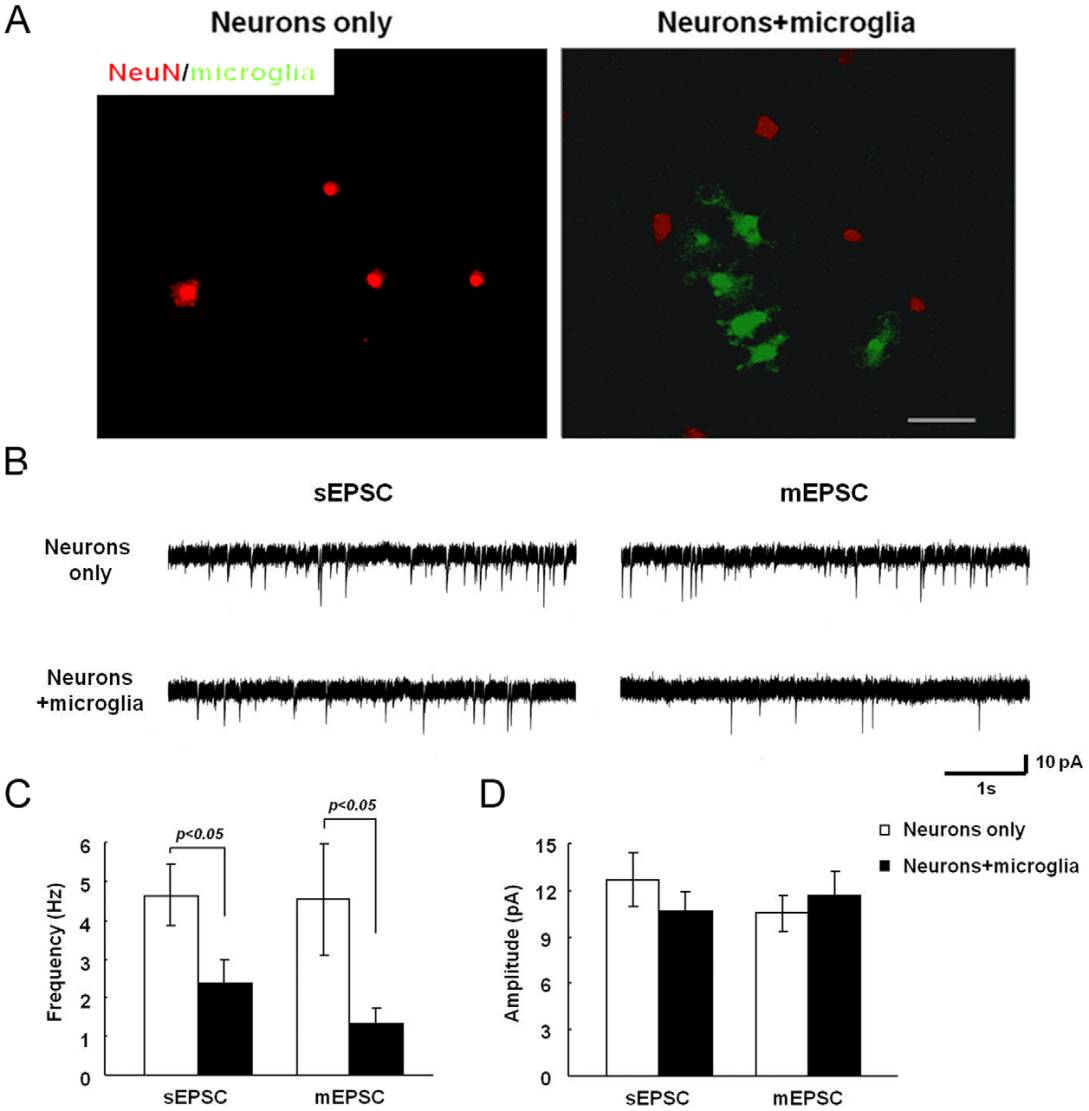
Addition of microglia to neurons reduces mEPSC frequency. Primary hippocampal neurons (DIV21) were cultured for 2 days either alone or in the presence of microglia obtained from MacGreen mice. **A.** Neurons were stained with anti-NeuN antibody and visualized with Alexa Fluor555-conjugated secondary antibody. Scale bar, 50 µm. **B.** mEPSC recordings from neurons in the presence or absence of microglia. **C, D.** Summary of mean mEPSC frequency (**C**) and amplitude (**D**) from neurons in the presence or absence of microglia.

To complement the results in organotypic slices, we characterized the anatomical and electrophysiological properties of primary hippocampal neurons cultured for 2 days either alone or with microglia at a ratio of 1 microglial cell to 5 neurons to mimic physiological conditions in the brain [19], [20]. Under these conditions, microglia remained in a resting morphology (Fig. **4A** and Fig. **S3**). The intrinsic properties of the hippocampal neurons showed no difference when cultured alone or with microglia (**Table 3**).

**Table 3:** Membrane properties of hippocampal pyramidal neurons in co-cultures with or without microglia.

Parameter	Neurons only	Neurons+microglia
RMP (mV)	−70.3±1.6	−73.4±2.0
Cm (pF)	19.8±1.5	17.0±0.9
Rm (mΩ)	344±24	336±33
Tau (ms)	6.1±0.8	6.0±0.6
Rheobase (pA)	232±22	311±32*
Time to the first AP (ms)	692±62	341±88*
AP peak (mV)	28.6±1.4	26.5±2.8
Threshold (mV)	−28.5±1.8	−21.5±2.2
AP half-width (ms)	2.0±0.1	1.9±0.2
AHP peak (mV)	−21.8±0.9	−23.5±2.0
n	27	15

All values are means ± SEM.

* , Statistically significant differences at p<0.05 when compared with neurons only.

RMP, resting membrane potential; Cm, membrane capacitance; Rm, membrane resistance; AP, action potential; AHP, afterhyperpolarization.

Complementing the data from the microglia-depleted slices, hippocampal neurons cultured with microglia showed significantly lower mEPSC frequencies (1.3±0.4 Hz, n = 16) in comparison to neurons cultured without microglia (4.5±1.4 Hz, n = 12; p<0.05; Fig. **4B, C**), or neurons exposed to clodronate (3.08±0.9 Hz, n = 2; p = 0.693), with no significant changes in mEPSC amplitude (neurons only: 10.5±1.2 pA; neurons+microglia: 11.7±1.5 pA; Fig. **4D**; neurons plus clodronate: 6.2±1.3 pA). These experiments further suggest that microglia regulate the number of functional excitatory synapses.

### Microglia regulate glutamatergic synaptic numbers

**Figure 5:**
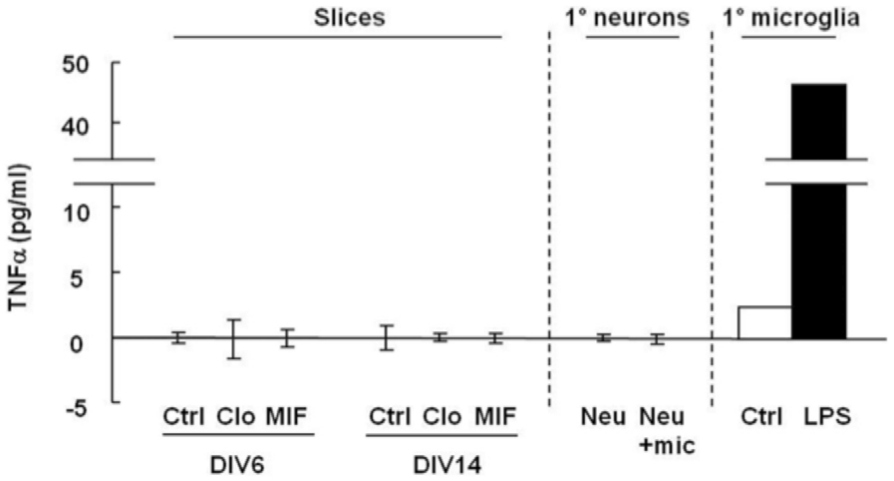
TNF-α levels are below detection levels in control or clodronate- or MIF-treated organotypic slices or in co-cultures of neurons and microglia. Media from control or clodronate- or MIF-treated organotypic slices at DIV6 and 14, and hippocampal neuronal cultures (1°neurons) in the absence (Neu) or presence (Neu+mic) of microglia for 2 days were collected. TNF-α levels were measured by ELISA. TNF-α levels in media collected from primary microglia exposed for 12 h to LPS (100 ng/ml) were used as a positive control.

Release of the cytokine TNF-α from glia, particularly astrocytes, has been reported as a potent way to enhance synaptic strength by increasing the cell surface expression of AMPA receptors in both cultured neurons and brain slices [21], [22]. We examined therefore whether microglial depletion triggers TNF-α release by the non-ablated glial cells. However, we found only very low levels of TNF-α in the cultured slices in the presence or absence of clodronate or MIF at DIV6 and 14, and in neuronal cultures with or without microglia (Fig. **5**), suggesting that TNF-α release by other cell types is not an important contributor in regulation of synaptic strength by microglia under physiological conditions.

**Figure 6:**
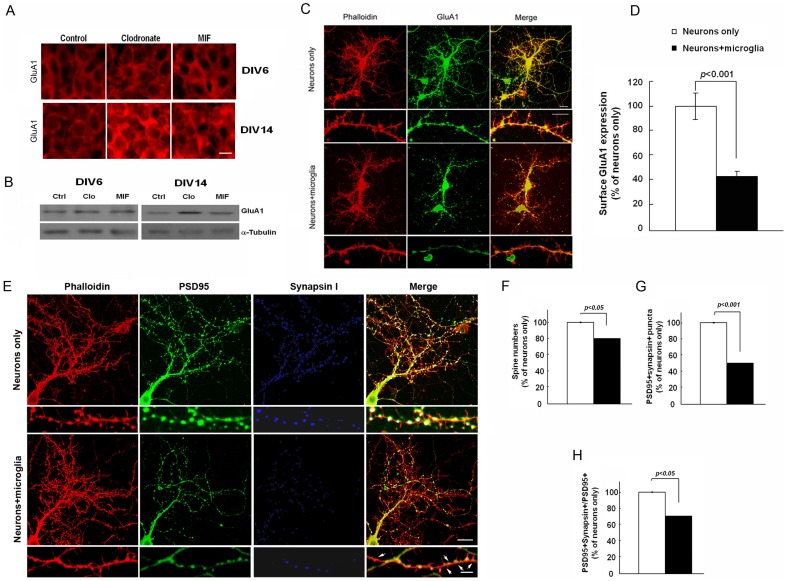
Microglia alter synaptic density and GluA1 expression in hippocampal neurons. **A, B.** Hippocampal slices were treated with clodronate (5 µg/ml) or MIF (100 µg/ml) for 6 or 14 DIV. **A,** GluA1 expression in the CA1 neuronal layer in DIV6 and DIV14 hippocampal organotypic brain slices. **B,** Lysates from the organotypic slices were immunoblotted and probed using antibodies against GluA1 and α-tubulin. Scale bar, 10 µm. **C, D.** Primary WT hippocampal neuronal cultures were cultured in the absence or presence of microglia for 2 days. **C,** Immunohistochemistry was performed using anti-GluA1 antibody under non-permeabilized staining conditions and visualized with Alexa Fluor488-conjugated secondary antibody. After subsequent permeabilization phalloidin was used to stain F-actin. Scale bar, 20 µm. **D,** Quantitative analysis of surface GluA1 along dendrites for neurons with or without microglia using the LSM 5 Image Browser (Zeiss). Data are presented as mean ±SEM and expressed as a percent of the neurons-only control sample. **E–H.** **E,** Hippocampal neurons with or without microglia were stained with PSD95 (green), synapsin I (blue), and phalloidin (red). The smaller boxes show magnified images. Arrows depict PSD95^+^ synapsin I^−^ puncta. Scale bars, 20 µm (upper panel); 5 µm (lower panel). Quantification of spine numbers (**F**), PSD95^+^ synapsin I^+^ puncta (**G**), and PSD95^+^synapsin I^+^ puncta in total PSD95^+^ puncta (**H**) in neurons cultured with or without microglia. Values are presented as mean ±SEM expressed as a percent of the neurons-only control sample.

To further assay the role of microglia in regulating synapses, we used immunohistochemistry and Western blot analysis to evaluate the expression of GluA1. GluA1 expression was increased in clodronate-treated brain slices in comparison to control and MIF-treated slices at DIV14, but not at DIV6 (Fig. **6A, B**). Consistent with this finding, surface GluA1 expression at dendrites was increased in neurons cultured alone in comparison to ones co-cultured with microglia (neurons only, 100.0±10.8; neurons+microglia, 43.0±5.3% of neurons only; p<0.001; Fig. **6C, D**) while there was no significant difference in the total GluA1 levels as determined by immunohistochemistry of permeabilized cells (data not shown). Similarly, there was no difference in the levels of GluA2 (Fig. **S4**) and GluN1 (data not shown).

**Figure 7:**
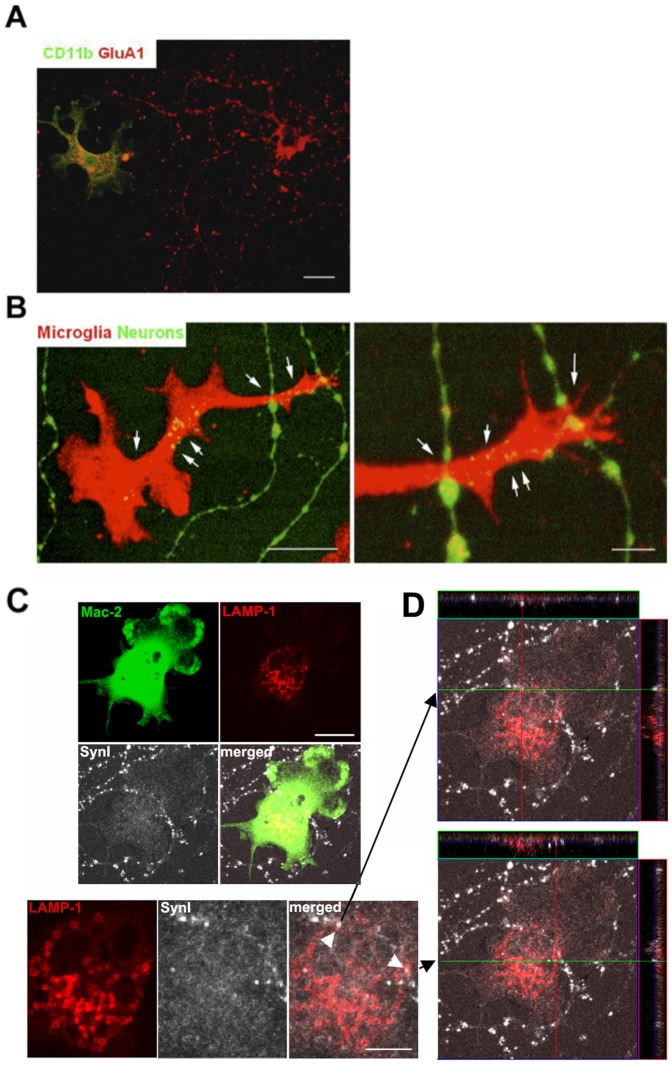
Microglia near neurons in co-cultures engulf neuronal materials. **A.** Hippocampal neurons 19 DIV were coincubated with microglia for 2 days, immunostained with CD11b (green), a marker of microglia, and GluA1 (red), and visualized with Alexa Fluor488 or 555-conjugated secondary antibodies. Scale bar, 20 µm. **B.** Images showing microglia (phalloidin, red) and neurons (obtained from β-actin-EGFP mice, green) in co-cultures. Arrows point to neuronal material within the microglia. Scale bars, 20 µm (left); 5 µm (right). **C.** Images showing Mac-2^+^ microglia (green), the lysosomal marker LAMP-1 (red), and presynaptic puncta immunostained for synapsin I (white) in co-culture. Scale bars, 20 m. **D.** Orthogonal views of the engulfed presynaptic material (white arrowheads in **C**).

We next examined synaptic density in primary hippocampal neurons cultured in the absence or presence of microglia for 2 days. Cells were immunostained with antibodies against synapsin I and PSD95, a presynaptic and a postsynaptic marker, respectively, as well as with phalloidin, which visualizes the actin cytoskeleton. Neurons incubated with microglia exhibited a significant decrease in the numbers of PSD95^+^ synapsin I^+^ puncta (neurons only, 100.0±0.4%, n = 27; neurons+microglia, 50.5±0.2% of neurons only, n = 39; Fig. **6E, G** **,** **Table 4**) and dendritic spines (neurons only, 100.0±0.2%; neurons+microglia, 81.0±0.2% of neurons only; Fig. **6E, F** **,** **Table 4**) compared to cultures of neurons alone. Synapses remaining on neurons in the presence of microglia contained less synapsin I than did neurons cultured alone (neurons only, 100.0±0.8%; neurons+microglia, 70.6±0.6% of neurons only; **Fig. 6E, H** **,** **Table 4**), suggesting that microglia contribute to regulating the numbers of presynaptic terminals. In these co-cultures, we observed that microglia were in the proximity of neurons using 3D reconstruction analysis; moreover, neuronal materials were engulfed by microglia (**Fig. 7A, B**). In neuronal-microglial co-cultures, presynaptic punctae identified by synapsin I staining were visualized in proximity with microglial LAMP-1, indicating that microglia were actively phagocytosing the punctae (**Fig. 7C–D**).

**Table 4:** Density of dendritic spines, PSD95^+^, synapsin I^+^ punctae in hippocampal pyramidal neurons in co-cultures with or without microglia.

Parameter	Neurons only	Neurons+microglia
Dendritic spines	4.8±0.2	3.9±0.2*
PSD95^+^	8.9±0.8	7.9±0.6*
PSD95^+^SynapsinI^+^	7.0±0.4	3.5±0.2*
n	27	39

All values are means ± SEM per 10 µm.

* , Statistically significant differences at p<0.05 when compared with neurons only.

**Figure 8:**
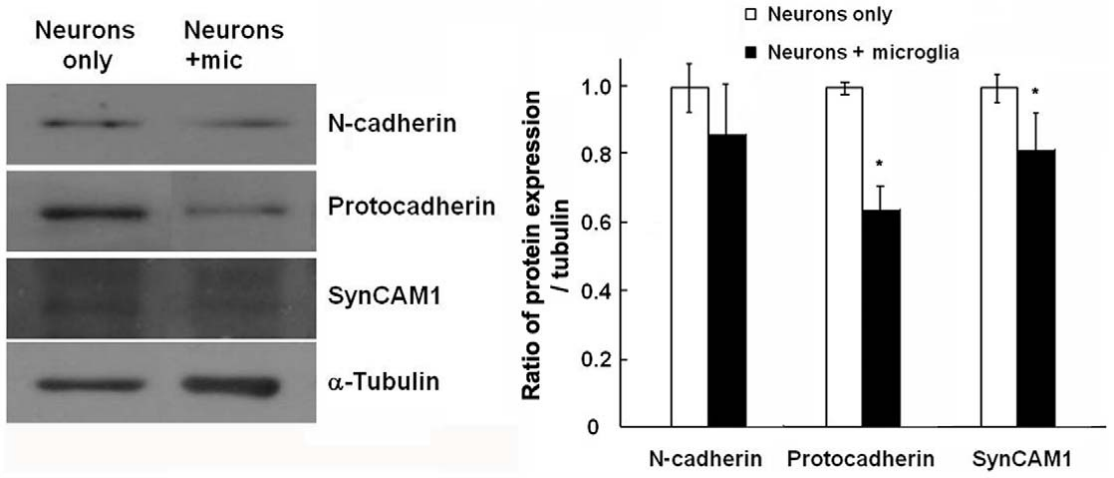
Microglia decrease the levels of synaptic adhesion molecules. Hippocampal neurons at 19 DIV were co-cultured with microglia for 2 days. **A.** Western blot analysis of the levels of N-cadherin, pan-γ-protocadherin, and SynCAM-1 in neurons in the absence or presence of microglia. α-tubulin was used as a loading control. **B.** Quantification was performed using the ImageJ software and normalized against α-tubulin (n = 4). *P<0.05 compared to neurons alone.

The stability of synapse spines and PSD95 depends on the expression of a specific set of synaptic proteins. Among these, it has been shown that adhesion molecules, in particular the N-cadherin-β-catenin complex, have a large role in synaptic strength regulation, late-phase long-term potentiation (LTP), spine stability and PSD95 expression [23]–[25]. Blocking of N-cadherin function in cultured hippocampal neurons alters dendritic spine morphology and disrupts pre- and post-synaptic proteins, suggesting that N-cadherin regulates dendritic spine morphogenesis and related synaptic functions [26]. We evaluated the protein levels of select adhesion molecules to examine if they would be affected by the presence or absence of microglia in neuronal-microglial co-cultures. As shown in **Fig. 8** in cultures of neurons alone, the expression of synCAM-1, N-cadherin and protocadherin can be readily detected. However, additional of microglia to the neuron decreased significantly the levels of synCAM-1 and protocadherin.

Taken together, these findings suggest that microglia actively regulate glutamatergic synaptic strength by modifying synaptic density.

## Discussion

In this study, we provide several pieces of evidence to support the hypothesis that microglia mediate changes in synaptic activity, and do so through involve altering synaptic numbers and glutamate receptor expression. Specifically, we show that (1) AMPAR-mediated mEPSC frequency is enhanced in microglia-depleted hippocampal slices and this increase is reversed by microglia replenishment; (2) Microglia addition to hippocampal neuronal cultures decreases AMPAR-mediated mEPSC frequency; (3) Changes in AMPA receptor 1 (GluA1) expression and functional synaptic numbers are consistent with the change in mEPSC frequency; and (4) The levels of synaptic adhesion molecules such as SynCAM-1 and protocadherin in neurons decrease when neurons are cultured with microglia. These findings suggest that normal, resting microglia interact with neurons in the normal brain with physical and functional consequences.

Previous work has demonstrated that astrocytic processes interact with synapses and regulate them by several mechanisms including glutamate transport and release of cytokines. Among them, TNF-α is known to increase synaptic strength in cultured neurons through TNF receptor 1 and AMPAR [21], [22]. However, our current data suggest that TNF-α is not the effector of synaptic changes in this physiological setting, since the TNF-α levels detected in our cultures are negligible. In another recent report, activated microglia were shown to regulate neurotransmission [27]. However, in this study, the microglia were shown to function indirectly and were termed as ‘upstream partners of astrocytes’. Our results indicate instead a direct effect by microglia on neurons, as in our culture system astrocytes were absent. Moreover, use of MIF to block microglial activation did not result in increased neurotransmission, indicating that the effect microglia have on synaptic activity is not mediated by activated microglia, but rather by resting microglia.

One consequence of the treatment with clodronate is that the CNS tissue is depleted of microglia and thus loses its primary phagocyte. Secondary phagocytic action is performed normally by astrocytes. Astrocytes do not seem affected by the treatments on the slices and therefore it is anticipated that their phagocytic properties remain intact. Although there was no effect of the presence of cell debris in the slice culture system, it is possible that the presence of microglia debris in the intact brain may result in localized inflammatory reactions. Moreover the absence of primary phagocytic activity in the intact CNS may lead to susceptibility to infections in the animals (persistence of CNS invasive infectious materials which are not removed) and to increased neuronal activity (as synapses are suboptimally pruned).

We report that in the presence of microglia, the expression of GluA1 and synaptic numbers are modified, and these changes lead to change in synaptic activity. Our work is consistent with previous results that showed that microglia participate in synaptic remodeling by engulfing synaptic components and the complement receptor 3(CR3)/C3 system during development [8]. Besides the complement pathway, microglial proteases alter the extracellular environment during physiological and pathological conditions of CNS. Proteases such as MMP-24 and ADAM 7 reduce levels of PSD-95 and AMPAR GluA2 and thus decrease mEPSC amplitude as well as synaptic vesicle reuptake, lowering mEPSC frequency [28]. Conversely, inhibition of protease activity increases mEPSC frequency and amplitude [28]. While direct cleavage of AMPAR subunits by microglia proteolytic enzymes could account for the differences in synaptic strength we observe, the more likely explanation is that microglia-mediated proteolysis affects synaptic stability. Schafer et al. [8] showed recently that microglia play a critical role in synaptic pruning during postnatal development via CR3 (complement receptor 3)/C3-dependent signaling. Moreover, we also showed that microglia in the proximity of neurons are able to phagocytose synaptic material, suggesting that microglia could regulate synaptic activity by engulfing synaptic components. Based on these findings, manipulation of proteolytic activities or components of the Cr3/C3 pathway in microglia could potentially be used to regulate synaptic strength.

In conclusion, our findings show that microglia regulate synaptic numbers and glutamate receptor expression, hence, they are directly involved in the modulation of synaptic activity. Moreover, the change in synapse numbers does not merely take place during development, but rather constitutes a dynamic, reversible, and continuous event in the mature brain. The action of microglia is acute and on-going in the mature CNS, and functionally significant.

## Supporting Information

Figure S1 **Clondronate ablates microglia in organotypic hippocampal brain slices.** Organotypic slices from MacGreen mice were treated with clodronate (5 µg/ml) or MIF (100 µg/ml) for 14 days and examined using confocal microscopy. They were stained with anti-NeuN (top) or –GFAP (bottom) and visualized with Alexa Fluor 555-conjugated secondary antibody. MacGreen (middle, green) indicates microglia. Scale bar, 10 µm for NeuN; 50 µm for MacGreen; 20 µm for GFAP.
(TIF)

Figure S2 **Clodronate treatment of organotypic slices results in cell death of microglia.** Organotypic slices from MacGreen mice at DIV14 were treated with vehicle, clodronate or MIF and stained with propidium iodide to detect cell death. Top panels show EGFP+ microglia. Bottom panels show propidium iodide staining. Scale bar 100 µm.
(TIF)

Figure S3 **Characterization of the state of microglia.** The diameter and number of primary processes emanating from the cell body were quantified for control and MIF-treated microglia, as well as for the exogenous microglia that were added to clodronate-treated slices. At least 5 cells were counted for each condition. No significant differences were measured in the different conditions (p = 0.21 for cell body measurements; p = 0.58 for number of primary branches).
(TIF)

Figure S4 **GluA2 expression of CA1 neuronal layer in hippocampal organotypic brain slices.** Hippocampal slices were treated with clodronate (5 µg/ml) or MIF (100 µg/ml) for 14DIV in vitro and stained with NeuN (red) and GluA2 (green).
(TIF)
