# Supplementary figures and images for: Microglia Actively Regulate the Number of Functional Synapses
Source: PLoS One. 2013 Feb 5;8(2):e56293. doi: 10.1371/journal.pone.0056293 (PMC3564799; doi:10.1371/journal.pone.0056293)

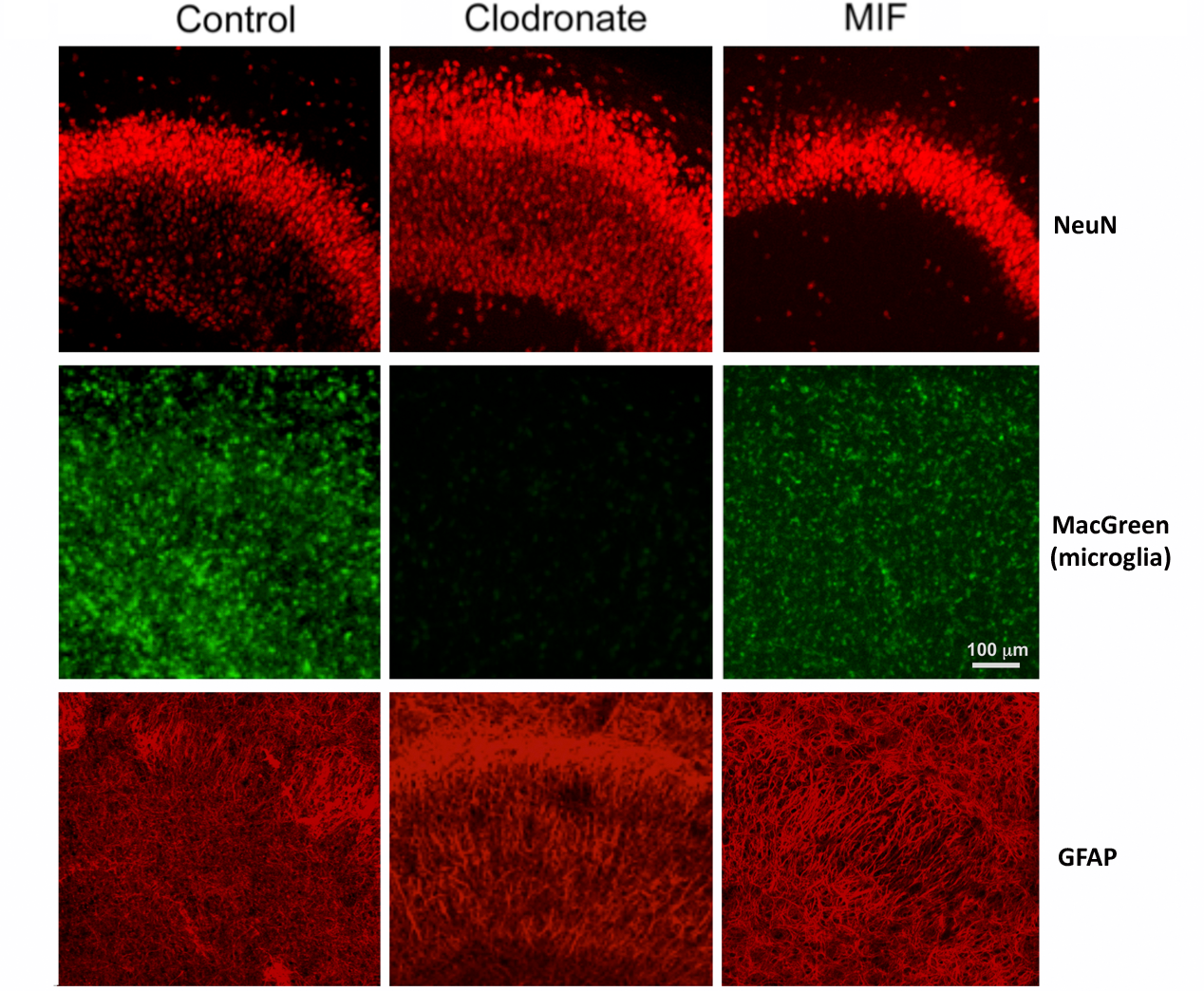

Supplement: Figure S1 — Clondronate ablates microglia in organotypic hippocampal brain slices. Organotypic slices from MacGreen mice were treated with clodronate (5 µg/ml) or MIF (100 µg/ml) for 14 days and examined using confocal microscopy. They were stained with anti-NeuN (top) or –GFAP (bottom) and visualized with Alexa Fluor 555-conjugated secondary antibody. MacGreen (middle, green) indicates microglia. Scale bar, 10 µm for NeuN; 50 µm for MacGreen; 20 µm for GFAP. (TIF) [file pone.0056293.s001.tif]

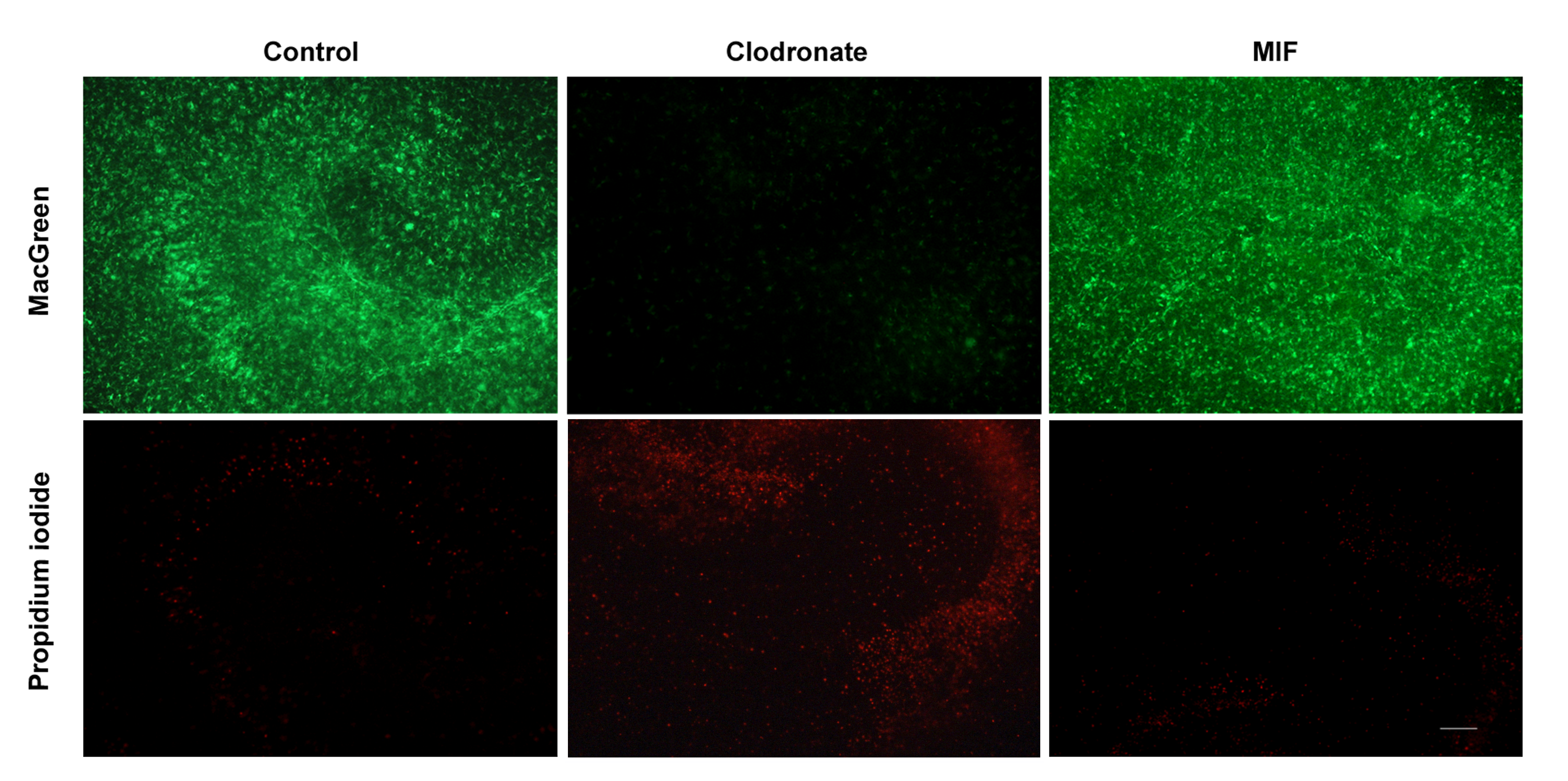

Supplement: Figure S2 — Clodronate treatment of organotypic slices results in cell death of microglia. Organotypic slices from MacGreen mice at DIV14 were treated with vehicle, clodronate or MIF and stained with propidium iodide to detect cell death. Top panels show EGFP+ microglia. Bottom panels show propidium iodide staining. Scale bar 100 µm. (TIF) [file pone.0056293.s002.tif]

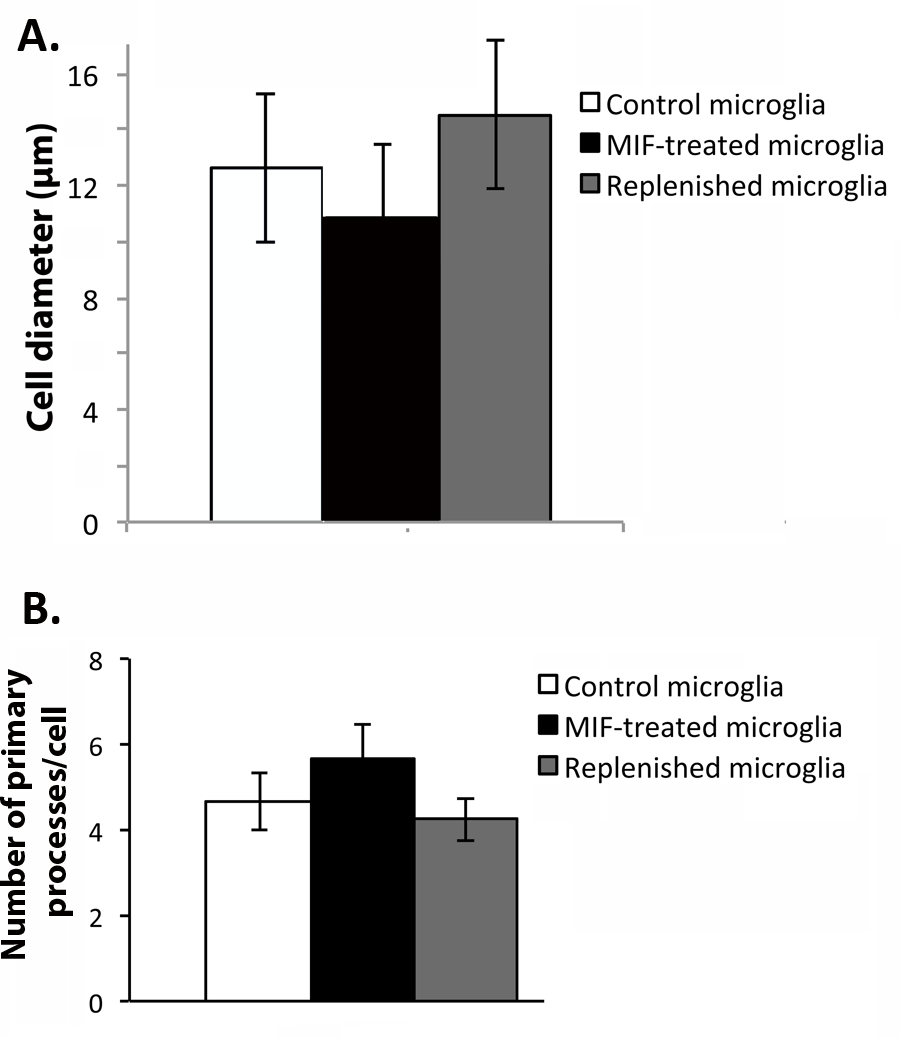

Supplement: Figure S3 — Characterization of the state of microglia. The diameter and number of primary processes emanating from the cell body were quantified for control and MIF-treated microglia, as well as for the exogenous microglia that were added to clodronate-treated slices. At least 5 cells were counted for each condition. No significant differences were measured in the different conditions (p = 0.21 for cell body measurements; p = 0.58 for number of primary branches). (TIF) [file pone.0056293.s003.tif]

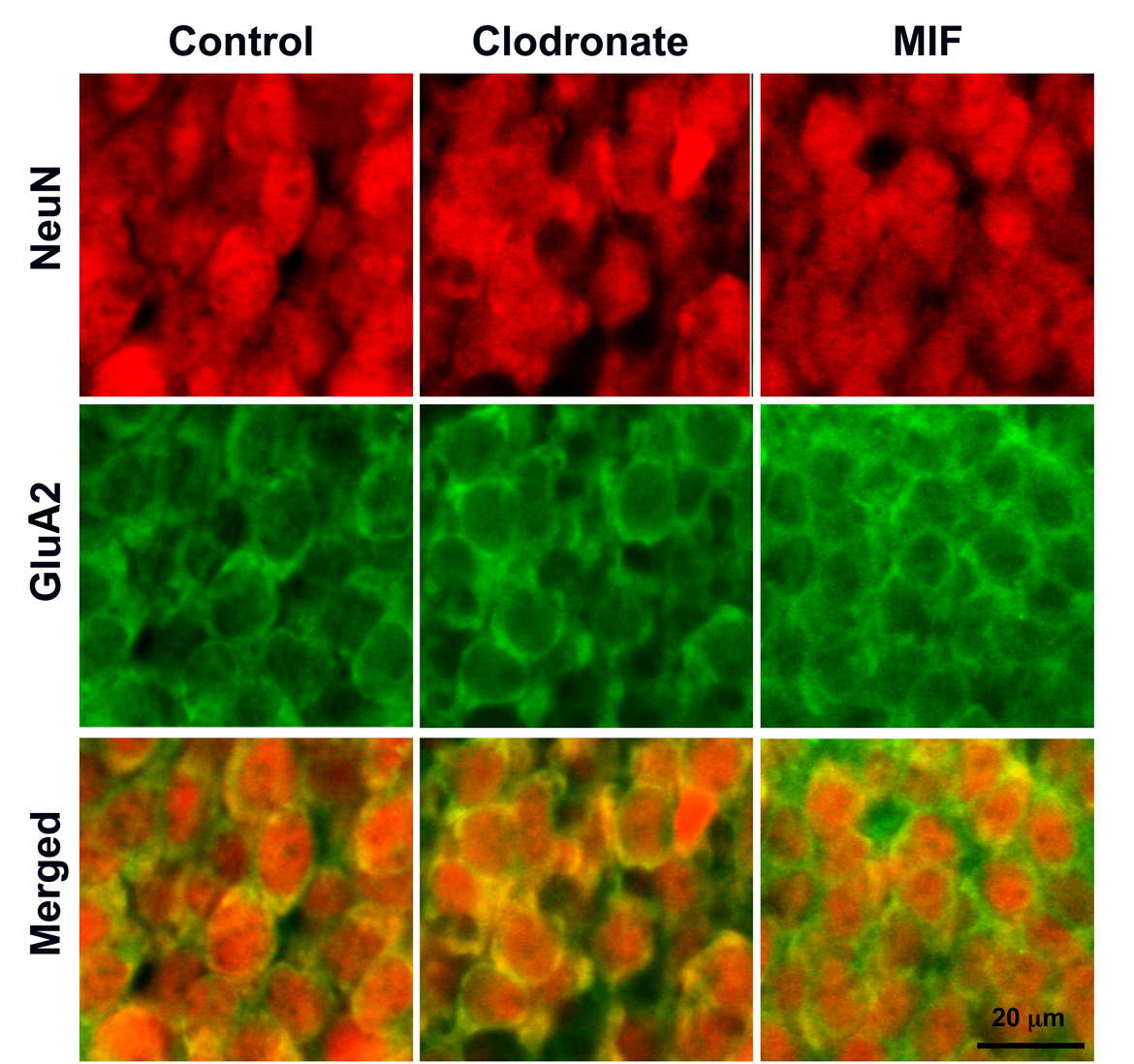

Supplement: Figure S4 — GluA2 expression of CA1 neuronal layer in hippocampal organotypic brain slices. Hippocampal slices were treated with clodronate (5 µg/ml) or MIF (100 µg/ml) for 14DIV in vitro and stained with NeuN (red) and GluA2 (green). (TIF) [file pone.0056293.s004.tif]
